# ‘Working to stay healthy’, health-seeking behaviour in Bangladesh’s urban slums: a qualitative study

**DOI:** 10.1186/s12889-019-6750-0

**Published:** 2019-05-17

**Authors:** Jeroen van der Heijden, Nell Gray, Beverly Stringer, Aminur Rahman, Sadika Akhter, Stobdan Kalon, Martins Dada, Animesh Biswas

**Affiliations:** 1 Médecins Sans Frontières OCA, Plantage Middenlaan 14, 1018 DD Amsterdam, Netherlands; 2 Manson Unit, Médecins Sans Frontières, Chancery Exchange, 10 Furnival St, London, EC4A 1AB UK; 3 International Centre for Diarrhoeal Disease Research, 68, Shaheed Tajuddin Ahmed Sarani, Mohakhali, Dhaka, 1212 Bangladesh; 4 Centre for Injury Prevention and Research Bangladesh (CIPRB), House B 162, Road 23, New DOHS, Mohakhali, Dhaka, 1206 Bangladesh

**Keywords:** Slum, Urban, Health, Worker, Sexual and reproductive health, Health-seeking behaviour

## Abstract

**Background:**

Kamrangirchar and Hazaribagh are the largest slum areas in Dhaka, Bangladesh. In 2013, Médecins Sans Frontières initiated an urban healthcare programme in these areas providing services for factory workers and responding to the sexual and reproductive health needs of young women. Little in-depth information is available on perceptions of health and health seeking behaviour in this population. We aimed to provide a better understanding of community perceptions toward health and health care in order to inform programme strategies.

**Methods:**

In-depth interviews were conducted with women (*n* = 13); factory workers (*n* = 14); and key informants (n = 13). Participants were selected using purposive maximum variation sampling and voluntarily consented to take part. Topic guides steered participant-led interviews, which were audio-recorded, translated and transcribed from Bangla into English. By comparing cases, we identified emerging themes, patterns and relationships in the data. NVivo11© was used to sort and code the data.

**Results:**

Emerging themes indicated that in Kamrangirchar and Hazaribagh, health is seen as an asset necessary for work and, thus, for survival. Residents navigate a highly fragmented health system looking for ‘quick fixes’ to avoid time off work, with the local pharmacy deemed ‘good enough’ for ‘common’ health issues. Health care seeking for ‘serious’ conditions is characterised by uncertainty, confusion, and unsatisfactory results. Decisions are made communally and shaped by collective perceptions of quality care. People with limited socio-economic capital have few options for care. ‘Quality care’ is perceived as comprehensive care ‘under one roof,’ including predictive biomedical diagnostics and effective medication, delivered through a trusting relationship with the care provider.

**Conclusions:**

Health seeking behaviour of slum dwellers of Kamrangirchar and Hazaribagh is based on competing priorities, where quick and effective care is key, focussed on the ability to work and generate income. This takes place in a fragmented healthcare system characterised by mistrust of providers, and where navigation is informed by word-of-mouth experiences of peers. Improving health in this context demands a comprehensive and integrated approach to health care delivery, with an emphasis on rapid diagnosis, effective treatment and referral, and improved trust in care providers. Health education must be developed in collaboration with the community to identify knowledge gaps, support decision-making, and be channelled through existing networks. Further research should consider the effectiveness of interventions aiming to improve the practice of pharmacists.

**Electronic supplementary material:**

The online version of this article (10.1186/s12889-019-6750-0) contains supplementary material, which is available to authorized users.

## Background

Rapid urbanisation is one of the most influential socio-economic changes of the last fifty years [1]. For the first time in history, more than half of the world’s population live in urban areas, with over a third thought to live in slum conditions characterised by poverty, poor housing, high population densities, and limited access to basic infrastructure and services [1–3]. In countries urban growth continues alongside rapid industrialisation and the expansion of informal economies; ‘slums tend to form the epicentre or principal source of informal labour, and within slums most economic activity is informal’ [1, 4].

Dhaka City, Bangladesh, is one of the largest and fastest growing cities in the world [3]. Approximately 35% of Dhaka’s population of 15 million people are thought to live in slums, which continue to expand as Dhaka’s population rises to an estimated 20 million by 2020 [3, 5]. Kamrangirchar is the largest slum area in the Dhaka city [6, 7]. Combined with the neighbouring area of Hazaribagh, it has an estimated population of 485,000 residing in approximately 6.5 km^2^ and is home to much of the city’s informal manufacturing industry, including an estimated 150 tanneries^1^ [6, 7].

In 2013, Médecins Sans Frontières (MSF) started an urban healthcare programme in Kamrangirchar and Hazaribagh responding to unmet sexual and reproductive health needs amongst girls and young women aged 10–19 years, and high rates of occupational illness and injury amongst workers in the area’s small-scale factories.

Previous studies of health seeking behaviour amongst Dhaka’s urban slum population suggest that the use of ‘traditional’ medicine has decreased as the use of biomedical services has increased [8–11], and that pharmacies are the principal healthcare provider for the majority of the population [12, 13]. However, as such studies are largely quantitative, little in-depth information is available to shed light on how and why such health seeking choices are made. Our objective was to document how people perceive their health and care options and seek healthcare within this community. By understanding gaps, barriers and factors that influence healthcare choices, we hope to inform MSF programming and strategy, and contribute to policy development for this and other similar vulnerable communities.

This qualitative study was conducted between March and June 2016 by MSF, in collaboration with the International Centre for Diarrhoeal Disease Research, Bangladesh (icddr,b) and the Centre for Injury Prevention and Research, Bangladesh (CIPRB).

## Methods

A qualitative descriptive explanatory approach was selected as most appropriate in meeting the study objectives; we aimed to both describe health seeking behaviour in this context (what is going on), and to explain factors influencing it (why is it going on)) [14]. As applied research directly linked to MSF programming, our sampling strategy focussed on the population for whom these services were relevant, comprising women and girls aged 13–49 years and factory workers. Key informants were also included, such as health workers and community leaders, to provide an additional perspective on health and solutions for ill-health within this community. Purposive maximum variation sampling was used to identify participants with a wide range of perspectives on the study aims in order to identify important shared patterns emerging from a heterogeneous participant group [15]. Individual characteristics considered in participant selection were gender, age, profession, geographical location (indicative of predominant industry, e.g. metal, leather, plastic, garments) and perceived vulnerability (based on housing type). Recruitment of the female and worker participant groups was facilitated by MSF project staff, who approached a group of dwellings and requested the participation of an individual meeting the inclusion criteria. Key informants were recruited using MSF team contacts. Data collection continued until the team felt that no major new information was emerging from interviews and so theoretical saturation was reached, comprising 13 to 14 interviews per group [16]. All participants took part on a voluntary basis and gave informed consent. We defined health-seeking behaviour as “…includ[ing] all those behaviours associated with establishing and retaining a healthy state, plus aspects of dealing with any departure from that state” [17].

Data was collected through in-depth interviews, using a flexible participant-led approach based on a topic guide (Additional File 1). Interviews took place in a mutually agreed private location, usually the participant’s home, and lasted for 60 to 90 minutes. They were conducted in Bangla or English, depending on participant preference, with JH and NG interviewing with the support of a translator. We ensured that the gender of the interviewers matched that of the participant. Interviews were audio-recorded, and recordings were transcribed and translated into English by experienced transcribers. Translators and transcribers received comprehensive training prior to data collection, as well as daily feedback from the researchers, JH and NG, to ensure careful contextual translation of idioms, metaphors, and other local expressions. Completed transcriptions were checked and a subset were back-translated by a second translator. Observations were written into field notes to support the interview data.

Data analysis was inductive and thematic using elements of phenomenological and grounded theory. This approach was selected as we aimed to generate new understandings of our study subject grounded in the views of our participants [18] and articulated through a descriptive narrative [19]. Open coding was used to break down, examine, compare, conceptualise and categorise data, followed by axial coding to ‘put the data back together’ in new ways, and selective coding to repeatedly apply core codes to transcripts (constant comparative analyses) toward the organic identification and development of patterns and themes emerging from the data [19]. Codes were subsequently gathered in conceptual categories and organised into themes through a process of analytic reflection [20]. Data were triangulated in order to maximise validity, and cases that did not fit with conclusions were re-analysed in order to test emerging theory and ensure that examples were not selected purely to reiterate desirable conclusions [21]. Data analysis was conducted by JH using NVivo11©, and a subset of data was coded and analysed by a second researcher, NG, to enhance reliability. Analytic memos were used to document the coding process and choices.

## Results

Interviews were conducted with 40 participants: 14 factory workers, 13 women and girls aged 13–49 years and 13 key stakeholders. Twenty participants were male (50%), 20 were female (50%); all were aged between 13 and 70. Table 1 provides an overview of participant characteristics.

**Table 1:** Participant characteristics

Participant group	Gender		Age group		
	Male	Female	13–17	18–30	31+
Factory workers (*n* = 14)	9	5	2	8	4
Female (*n* = 13)	0	13	4	5	4
Key informants (*n*-13)	11	2	0	8	5
Totals (*n* = 40)	20 (50%)	20 (50%)	6 (15%)	21 (52.5%)	13 (32.5%)

Over 80 codes were gathered into nine conceptual categories organised in three themes: 1) sustaining life and health: the responsibility of staying well; 2) competing priorities and fragmented health care: how decisions are made; and 3) quality care: quick effective medicine, trust and a comprehensive approach.

### Theme one: sustaining life and health: the responsibility of staying well

Many participants described personal or family narratives of relocation to Dhaka from rural areas, primarily motivated by the economic opportunities available in the city. They explained they wanted to escape rural poverty, increase earnings and improve their economic status.

*‘Here we are working hard and earning a bit to carry on our living expenses. The days are going well somehow. In the village, there was a constant feeling of wanting. We were compelled to poverty there. Here we are all working and living a good life.’ P18 - Housewife (HW).*

When discussing daily life in Kamrangirchar and Hazaribagh, most participants did not place an emphasis on health as a predominant concern. However, when specifically questioned, the majority stated that the general health status of the population was poor: *“People here, almost 90 out of 100 are sick.”* (P17 – Key informant (KI)).

Participants described three main interrelated factors they felt impacted their health: poverty, work, and the environment. These factors emerged as central to participants’ perceptions of ‘staying well’.

Earning enough money to make ends meet was the focus and preoccupation for the majority of our participants on a day-to-day basis. Many mentioned how their precarious financial situation shaped their priorities and decision-making, and ultimately had a negative impact on their health. This was largely expressed through the need to work long hours in poor environments, and difficulties affording the quantity, quality and variety of food necessary for good health.

*“If you want to eat healthy then you need money. You need it to have fish, meat to stay healthy. So where am I going to get money to buy this?” P3 - Male factory worker (MFW).*

Most participants worked in formal or informal small-scale factories producing garments, plastic, metal or leather goods. The majority of female participants not engaged in factory work undertook additional work at home as well as fulfilling household duties (handicrafts, tailoring, cooking etc). In many cases participants reported harsh working conditions, poor personal protection and long working weeks (generally twelve hours a day, six days a week). Work was perceived to have a direct negative influence on health, either due to workplace hazards or the sheer intensity of work and the lack of rest.

*“Hard working in poly [plastics] factory is the main responsible of health disease. I feel headache because of working. When I go to work, my headache starts instantly. My hard work is the main reason. Besides, I have to maintain my household work, my children. I work there twelve hours.” P6 – Female factory worker (FFW).*

*“Well, as I do the welding, it is seen that it harms the eyes. Due to welding, things seem to be blurred. Sometimes, due to excess working and sweating, I feel tired. As we do works with iron materials, so it’s normal that sometimes there are some accidents. Maybe there is a wound by hammer… Sometimes the wounds are so severe and painful. Sometimes the wounds don’t heal in proper time if there is an infection.” P13 - MFW.*

The environment in the area was perceived to be poor and have a negative effect on the health of its inhabitants, notably due to poor-quality water and pollution from the area’s factories.

*“Hazaribagh holds the tannery factories. It is very polluted. Whenever you go there, you will find an odour there. It smells very bad. People use handkerchief to protect their nose from this odour. Some people even vomit. So, the environment of Hazaribagh is very bad now.” P30 – KI.*

*“I think water is the main problem [causing sickness]. The water here is very polluted. We can’t even purify the water because it is polluted.” P22 - HW.*

Our participants emphasised that in this context *‘people consider their health as an asset’* (P01 - MFW) and the majority of participants defined ‘health’ and ‘ill-health’ in terms of their ability to work and maintain an income. People described illness as tolerable as long as they were able to work, and shared experiences of working with chronic pain or low-level health issues. Ill-health was considered particularly problematic when severe enough to prevent work. This perception of the ‘well body’ as a means to, and prerequisite for, subsistence also had a significant impact on health seeking behaviour.

*“Now most of the people are workers, they are working and eating and moving and maintaining their family, they work all day… We see that many people have to work a lot daily to run their family. They don’t think [of] their pain as pain.” P12 - KI.*

A sense of individual and collective responsibility for ‘staying healthy’ emerged amongst all participant groups. They described the importance of adhering to ‘rules and regulations’, both as an individual and a community, expressed as the importance of a balanced diet, environmental cleanliness and adequate rest.

*“You are responsible for your good health. You need to be careful about your health. God has bestowed knowledge upon you and you should use this knowledge. You are responsible for your health. None is guilty for your sickness or frail physique.” P17 - KI.*

*“If a person does work, takes rest and food in time, and then he or she can lead a healthy life.” P04 - (MFW).*

*“If all of us come together to keep the environment clean and to keep an eye on what are you eating, and if we eat healthier food then we can keep ourselves away from many diseases.” P01 - MFW.*

It was also evident that faith played an important role in how some participants perceived their health, explaining the ultimate responsibility for their wellbeing lay with god.

*“Health is something people pray for a sound well-being... I just pray to Allah the almighty to keep me healthy…. People do expect to live a healthy life and pray for his healthy life from Allah the almighty. Today I am here talking to you but there is no guarantee that tomorrow you would find me or not. But there is at least a hope.” P01 – MFW.*

### Theme two: competing priorities and fragmented health care: how decisions are made

A common health-seeking pathway emerged from participants' narratives. Many explained that self-care was the first step in restoring health, prior to seeking care from a provider. Rest, often combined with taking saline solution, vitamins, or eating extra or better food, was perceived as critical to recovery. When these actions were not feasible due to work commitments or a lack of financial resources, or did not work, many participants reported that a pharmacy was the first service sought to resolve most health issues.

*“I take rest, but when it doesn’t improve, the tiredness still remains then I go to the pharmacy and discuss.” P07 - MFW.*

*“Actually, as usual in Bangladesh, I went to a pharmacy, when I had the fever, when I felt a little weak, and brought some simple medicines. Almost everyone does the same to see whether they get well or not… 99% of people, if they encounter fever, cold, if the intensity is less, they go to the pharmacy and ask for medicines.” P37 - KI.*

If the desired results were not achieved, various alternative options were consulted, often in quick succession and with the expectation of seeing fast results; participants reported trying other medications, switching to a different pharmacy or seeking care at a higher level from a hospital or ‘proper’, ‘MBBS’ doctor.

*“Later when I observe the treatment of pharmacy wasn’t working well. Then I decided to go [Hospital A].” P6 - FFW.*

*“There are many big medical and hospitals surrounding Hazaribagh. People go there [when] severely sick otherwise take medicine from pharmacy. If one medicine doesn’t work, then switch on to another medicine.” P19 - MFW.*

Whilst a general preference for allopathic care was evident, some participants mentioned people fell back on alternative practitioners such as a *hakim* (herbalist) or *kobiraj* (spiritual healer) for a number of reasons, often when other medication failed or finances ran out.

*“I had medicines from the pharmacy for this problem as well. But it didn’t work. I also had antibiotic. Then they suggested me to go to [Hospital A]. Then we thought it would cost us a lot of money but homeopathy is much cheaper than this. I later on went to the homeopathy and got a good result.” P22 - HW.*

*“They go to doctors and hospitals. Try modern medicine. When all fails then they come to herbs. People now do not believe in herbal treatment…. whatever may be the sickness is it is important for the patient. Therefore, he would go to the option what he thinks is the best for him. If someone comes to me [a herbalist] with a headache and I can’t fix it then he will go to another doctor.” P17 - KI.*

An exception to this pattern was described by a minority of participants, who gave alternative explanations of disease requiring traditional, herbal or spiritual treatment.

*“…whenever we get headache [the kobiraj] comes to treat and it works… in daily life many people get caught by supernatural powers then he treats that and it works.” P09 - HW.*

Participants explained they also used care provided by non-governmental organisations (NGOs), specifically vertical services for family planning and reproductive health. Whilst perceptions were mixed, free and accessible care motivated uptake.

*“There is NGO A for maternity for the pregnancy issues, NGO B are also here. They also provide a good service… I take [family planning] services from NGO B, injections every three months. Earlier I used to take the same service from NGO D clinic because it was funded and maintained by international donors. But now as the service is switched to Bangladeshi management that is why I go to NGO B. It is totally free and operating in almost every area.” P22 - HW.*

Recounting these health seeking ‘journeys’, participants described a huge range of care providers, including NGOs, private clinics, government hospitals and alternative practitioners, each providing a partial service. For participants this was experienced as a fragmented and inconsistent ‘maze’ of options to be navigated by the patient. They told stories of bouncing from one provider to another in search of a solution, often getting lost, frustrated and confused by contradictory recommendations and diagnoses.

*“I have gone everywhere and now when the pain increases in my body I am just taking rest… and now I am not going anywhere… what will happen after taking [medication]? I don’t know. I never saw that or know anything about that... then he said I don’t need anything. Then he prescribed some medicines. I don’t go there after taking those medicines... I feel annoyed to go there and come back.” P09 - HW.*

Participants explained that decisions about where to seek care and when were based on careful calculations in terms of money and time. Lack of available funds often dictated choices, as participants described choosing the cheapest option, usually their local pharmacy. Generally, more expensive services were seen as ‘a last resort’, to be consulted when others failed. For some, cost rendered such options inaccessible. Financial constraints also resulted in delayed care-seeking and partial treatment, such as taking incomplete prescriptions or being unable to afford recommended diagnostic tests.

*“If we visit to the doctor, we need money first, but whatever we earn, we have to spend on our household expenses. I have sacrificed my life for my family… Everything depends on money. I have to run my family. But if I spend money for treatment then I have to think about next situation. That’s why I have to tolerate my sickness… When we feel fever, I talk with the pharmacist. He says to me that if you take this medicine you will be alright within 15 days. I follow their recommendation. When I feel well within 8 days, I just stop taking medicine for next few days which he recommended for 15 days. Because I don’t have enough money to buy more medicine. I have to think about money.” P05 - FFW.*

Time was also a critical factor shaping care choices; for this predominantly working population time and money were synonymous, so an emphasis was placed on a ‘quick fix’ to health problems. At the same time long waiting times acted as a disincentive to accessing higher level or hospital services.

*“We are poor. Time is very important to us. It takes huge time to visit [Hospital A] for treatment and we also need to stand in line for a long time to see the doctor… Due to the shortage of time I don’t have a choice, that’s why my first choice is pharmacy.” P05 - FFW.*

*“We are labourers; we can’t waste a day to go to the medical as we would lose a day’s income.” P34 - HW.*

Participants also mentioned the indirect and hidden costs of healthcare as a barrier to accessing services, notably to comprehensive secondary or tertiary care located outside Kamrangirchar. Distance was expressed in terms of travel time and costs, which were often prohibitive, providing another reason for participants to prioritise nearby care options.

*"I have to spend 100 taka* 2 *for transport [to get to the hospital]. On other hand, if I buy medicines that cost 100 taka, in that time I can be recover and I also can save my time. So with this problem we prefer to go to pharmacy directly." P05 - FFW.*

Perceptions of symptoms also informed actions taken to restore health, as participants explained they often self-diagnosed based on previous observations and experiences, and classified their illness as ‘common’ or ‘serious’. On the whole, people saw the pharmacy as a suitable option for ‘common’ diseases such as colds, coughs or headaches. Once a condition was thought to be more ‘serious’, a different care seeking avenue was chosen, generally involving more formal services, ‘MBBS’ doctors and a greater financial outlay.

*“I know it very well, if anybody of my family feels too much sick, we should visit to the doctor first. If situation is very bad then to the hospital, if not then I’ll go to local pharmacy.” P04 - MFW.*

Participants explained that health issues were considered ‘serious’ if they did not respond to pharmacy-bought medication; presented unfamiliar symptoms; and/or were serious enough to prevent day-to-day activities, specifically work. These illnesses were characterised by uncertainty and a lack of understanding, and the need for diagnostic tests *“to understand what is actually going on inside the body”* (P16 - FFW).

*“[A disease is serious] when it doesn’t become well after taking lots of medicine. Day after day the situation is getting worse, bones are bending face is bending…” P28 - FFW.*

*“It is very easy to understand that what is very common. But it is very difficult to understand a serious disease. If it is very serious or not understandable then I talked to sister or aunty. Then they go to the pharmacy to get details about the problem. Or do some diagnostic test to reveal what the matter is. By doing this we can measure is it serious health issue or not... I can only understand common problem like fever, headache. But I don’t understand any major problem. You can only understand major problems by doing some diagnostic tests like blood tests, urine tests etc.*” *P16 - FFW.*

The majority of our participants obtained knowledge about health and health services through word-of-mouth. Information spread through the community as people shared their knowledge and ideas and learned from past experiences. Women particularly described the central role of their social networks to inform decisions about health, seeking advice from trusted female friends and family members.

*“Yes I [take advice from friends or family when I’m ill]. I think that’s very normal. Whenever I don’t understand the problem then I usually ask my friends or relatives to help me out. Sometimes I take advice from them about how to take the pills and medicines.” P01 - MFW.*

*“I learn something from experience, like if someone caught fever or cold related problem they just take Napa tablet [paracetamol] for cure. Sometimes I also apply for this on my own problem. I saw my manager doing so when he feels sick; he just took some medicine like that.” P16 - FFW.*

For some, this information gathering process yielded contradictory advice, which increased confusion and uncertainty when facing numerous possible options for care.

*“[I] ask people, ‘where is the best place for treatment?’. There are big brothers and sisters [that] I can ask about this. […] Problem is many people advise differently. So it is difficult to judge which decision is best.” P16 - FFW.*

These informal channels also posed challenges to accessing information on sensitive health issues, as participants mentioned feeling shy or uncomfortable sharing their symptoms with others. This was noted particularly for girls and younger women linked to sexual and reproductive health and family planning:

*“Many girls cannot share what they should do after marriage due to shyness.”* *P18 - HW.*

*“It is better if I get more ideas [about family planning]. As it was [my pregnancy] happened by mistake, so if I knew it earlier then I would’ve not made that mistake or won’t make further. So I am in need of such ideas… I didn’t have any idea [about family planning] before I got married. I didn’t think of these things before... [After I was married] then also I didn’t get any ideas or information. If someone would have asked me about this then I would pretend that I don’t know about these things.”* *P27* *- HW.*

Many participants explained that health decision-making within families had undergone changes over time, with responsibility and authority no longer held soley by the head of household as it had been in the past. For the majority of participants, however, health decisions were made collectively and informed by the opinions of family members, partners, and in some cases employers. This was particularly evident for women; those who were married generally consulted with their husband when seeking care for themselves or their children, and younger girls deferred decisions to their parents or older female relatives. This was often linked to the financial implications of the care choice, as those living separately from their husband or earning independently expressed more autonomy in terms of choosing and accessing care.

*“There was once a time when the head of the family used to make decisions whether to go to hospital or not, will I take my son or daughter or wife to the hospital? There was once a time when we had to wait for his decision. Now the situation has changed. Now they are all getting to know from the welfare of different NGOs that they have this problem and they have to go to the hospital for treatment. Now the decision is being taken by every member of the family. Once it was like this that father-in-law used to be head of the family and to make decisions for taking to hospitals or not but now mother-in-law can also take the decision”. P24 - KI.*

*“It can be different in different families, but many times it just happens that the husband or the earning person take the decision where people, where the family goes for their health.” P26 - KI.*

*“It was my decision [to have an abortion]. I didn’t say this to my husband or to my mother-in-law.” P33 - FFW.*

For many participants, personal connections with care providers, as well as referrals from one trusted provider to another, often dictated the next step in a care-seeking journey. This was often linked to the perception that the referral would facilitate quicker, better quality or discounted treatment.

*“My sister in law works [in the clinic] so we visit privately the doctor works there. So we are his people. My sister in law directly talks with the doctor, then she took me there, then she listens to me, then he gave me treatment, the treatment he gave we took them immediately…” P28 - FFW.*

*“[The pharmacist] gives medicines if he can. And if it is not possible for him then he suggests us to visit the hospital. At least I get ideas what to do in various situations… They refer some doctor in the hospital if they know someone closely. They provide me small notes or cards and say I might get discount, quick and good support on medical facilities if I show the small notes provided by the pharmacist.” P01 - MFW.*

Participants explained that additional sources of health information comprised the many organisations going door-to-door, including people selling drugs and traditional remedies, and workers from various NGOs. Whilst some participants appreciated this effort, others expressed confusion at the different messages and services offered and mistrust in the motives of the workers.

*“They go door to door to seek people and patients, they come to sell medicine… they say that they are doctors: ‘don’t mistrust us, we stay with the doctors, see – we have uniforms… we know what to give for what diseases, we don’t want you to be sick, we want that you stay good, that’s why we come door to door with care’. They come house to house just to keep their jobs...” P28 - FFW.*

### Theme three: quality care: quick, effective medicine, trust, and a comprehensive approach

Participants expressed the importance of quality care, and they described a willingness to overcome significant barriers to access this. Participants noted three elements as indicating quality: effective and fast treatment; a trusting relationship with the provider; and the availability of comprehensive services.

For most participants, medicine and medical care were deemed to be effective because care seeking took little time and lead to a fast recovery.

*“[The service I like the most] is the one we have to give less time…” P09 - HW.*

*“I went to him [a qualified doctor] and he checked properly. He advised medicine for three days. But after taking medicine for one day my daughter was cured. He takes 300 taka as fees but he prescribes less medicine.” P20 - MFW.*

However, whilst all participants valued ‘quick fix’ medication, participants also raised concerns about the quality of drugs, and what to take when, as well as the risks linked to inappropriate prescribing, drug resistance and side effects. Several also saw the results provided by drugs as superficial or short-term, as a means of finding ‘*temporary relief*’ (P15 - FFW).

*“In fact, I hardly take medicine. Because if I take much medicine now then in older age it will be so painful. At that time medicines will not work well.” P17 - KI.*

*“I am newly married... it was seen that, [my wife] feels some pain. Then she doesn’t understand what to do. At times she has been given kind of worthless medicine for decreasing the pain. It doesn’t mean that this pain is curable with this medicine… Then she is saying that there is some pain, ok, bring a pain killer. Then, it is working; it is stopped for a few days. After a few months, again the same pain is arising. Thus, we are giving medicines without understanding. Now, if we could know what the problem is or if we could take better medication from a good doctor then it would be much better.” P13 - MFW.*

Exceptions to this pattern explained that they either did not like taking medication or preferred homeopathic or herbal remedies as they provided more long-term solutions, ‘*cure[ing] the problem from the root*’* (P22 - HW).*

*“I don’t take medicine; I don’t love to take medicine, that’s why I don’t go to the doctor… I’m afraid to take medicine from my childhood.” P04 - MFW.*

*“But people nowadays want everything to be fast. That is why they visit to the allopathic. On the other hand, if you consume allopath in the morning then you might get well in the evening. But a person has to consume homeopathy for at least a week to be well. But allopathic has side effects and doesn’t works extensively which homeopathy does.” P01 - MFW.*

The relationship between the practitioner and the patient was another significant factor in defining quality care for all participant groups. Many participants described negative experiences resulting from poor attitudes of health workers and a lack of time or ‘attention’. This was particularly evident in descriptions of busy government hospitals.

*“…when the baby is born they don’t take care. Like few days ago a kid was sick, had a fever, they went there [to the maternity clinic at [location A]] but they didn’t give any attention. After that they went to another clinic and brought medicine from there. I heard that from them; they said the baby was born there and had a [patient] card there but they didn’t him give any importance.” P28 - FFW.*

*“Doctors don’t have any personal time for the patient and it very simple because it’s a government hospital.” P05 - FFW.*

For several participants, negative attitudes of health workers were explained through the social distance felt between patients and providers; poor treatment was described as ‘normal’ given the poverty and ‘low status’ of Kamrangirchar and Hazaribagh residents.

*“Oh listen we are poor, people do they value us? They become irritated if we go for the second time. They don’t give importance. If they did then my treatment would be good…. They just become irritated and as we don’t have any education…” P09 - HW.*

Mistrust of providers was also evident and a deterrent to service uptake. This was largely based on the proliferation of unqualified ‘doctors’, and suspicions around the motivations of health workers perceived as using ‘healthcare as a business’, for example asking for bribes.

*“Many doctors become businessmen. For them, money is a bigger issue than a patient. They think like they I have become a doctor after going through so much sufferings, why shouldn’t I earn money. For this purpose they build a liaison, they build a relation, a mutual relation with those organizations who do the tests, with the conditions that- if you recommend these tests here, as long as the patients come here, you will get a percentage for that.” P37 - KI.*

Conversely, attentive care was very positively received by patients and was often described to justify paying more or travelling further. Building a relationship between the caregiver and patient and establishing confidence and trust were described as important to quality care. For some, the qualification of MBBS or costly care also instilled confidence as a guarantee of ‘genuine’ treatment, and so a quicker recovery.

*“My father has got some problem in his body… and my mother-in-law faces problems whenever she gets fever. They both consulted doctor in [Hospital C] in Dhanmondi nearby [Hospital D]. The service they got was excellent but the cost is higher. But in [Hospital A] the cost might be cheap, but the treatment is very poor.” P22 - HW.*

*“The pharmacists have very good relationship with the community people and they just are a familiar person.” P26 - KI.*

*“My experience [as a doctor] is that some patients are confused about doctors… If he is a doctor or not. Then after the treatment, if he or she feels better, or if my investigation, my drug, my behaviour, attitude, and his problem if solved… then he believes. After that, he receives my advice. Trust. Trust. First need trust.” P40 - KI.*

Lastly, for many participants comprehensive care was described as fundamental to perceptions of quality. This was described as facilities offering a complete package of services, including diagnostic tests and secondary level care, where patients could receive necessary consultations and treatment ‘under one roof’. This was identified as a critical gap in services available to Kamrangirchar and Hazaribagh residents, with an observable negative impact on health.

*“If there was a good hospital here they wouldn’t need to go anywhere else, […] like if there was something centralised here [in Kamrangirchar].” P36 - KI.*

*“I think if there is a hospital here then we general people will have a health facility and we can visit a good doctor. Now here all we have are unqualified doctors. The doctor is like us. Not hi-fi. Now if we get a fever we buy 10-taka Napa [paracetamol]. And if the fever doesn’t reduce then we have to go to the medical. A few days ago, the dengue mosquito bit some people and they died from the fever, because now the service is not here. If there is a medical, then we will benefit, if there is a doctor to give us tests. But now who will give us tests? And whom are we going to show the tests?” P03 - MFW.*

## Discussion

Three major themes emerged in relation to the perceptions of health and health services in Kamrangirchar and Hazaribagh: sustaining life and health; competing priorities and fragmented care; and quality of care.

Work assumes a central position in the life of residents of Kamrangirchar and Hazaribagh. Employment in the area’s informal industries represents an escape from rural hardship, a necessity for day-to-day survival, and the possibility of economic betterment. Our participants describe that they work to alleviate poverty, yet they do not achieve the economic freedom necessary to take measures to ‘stay healthy’: eating a balanced diet, accessing ‘proper’ healthcare, taking adequate rest, and ensuring a clean environment [22–24]. Long working hours and poor working conditions directly and indirectly impact health, causing illness and injury and limiting time available to recuperate or seek care. Furthermore, the expenses related to seeking care – for consultations, medication and other hidden and indirect costs [25] – are often unacceptable, and taking time off work problematic for fear of losing the day’s income and even the job [26]. Therefore a ‘status quo’ is established in which health is commodified, with the ability to earn being prioritised and ‘functional health’ (the ability to perform work roles in the short term) taking precedence over longer term concerns about wellbeing [27].

In this context, time is money, providing a ‘fertile ground for a flourishing trade in medical fixes’ [27], and an inexpensive ‘quick fix’ at the local pharmacy is generally the first step taken in restoring health. This pattern has been documented in previous studies conducted in Bangladesh and other low-income urban areas. It is thought to have a negative impact on health outcomes [8, 12, 28, 29], and be ultimately more expensive for the individual, the family [24, 29], and the health service [30]. For Kamrangirchar residents, however, pharmacy treatment is inexpensive, fast and close to home, restoring people to their productive role in the household with minimum disruption to the delicate balance of the family economy. However, faith in pharmacy treatment is varied; for some it provides just ‘temporary relief’, curing the symptoms but not the cause, and for others the risks of mis-prescription, resistance and side-effects are concerning. As a result, pharmacies are the first recourse in case of ill-health due to the need to maintain other priorities, rather than the quality of care provided or to an inherent value placed on medication. Several studies document this and call for increased regulation combined with training programmes directed towards drug sellers [31–34]. Evidence of the effectiveness of such interventions is limited, although previous studies suggest that it is possible to improve pharmacy practice in similar contexts with a combination of improved enforcement of regulation, education, and peer networks [35].

Decisions about health and care are determined by the perceived ‘commonness’ or ‘seriousness’ of a condition, based on individual and collective experiences of symptoms [31]. A ‘serious’ condition is identified when pharmacy treatment is ineffective, symptoms are unfamiliar, or are severe enough to prevent work. This triggers people to seek diagnosis and treatment from qualified doctors, often in secondary level facilities and supported by diagnostic tests. Significant barriers are overcome to access care in these circumstances, as also found in studies in India [28, 32], notably in daily wage-earning slum residents [33].

Our study illustrates that seeking care outside of the pharmacy setting involves negotiating a fragmented landscape of partial services, many of which are poorly regulated and provide care of varied standards, as described by other studies in urban slum settlements in Bangladesh [34], India [36] and elsewhere [23, 34, 37, 38]. Combined with inadequate provision of secondary and tertiary services [29], this is incompatible with a comprehensive health services approach and creates a high level of uncertainty for slum dwellers in relation to the availability and quality of care. People consult different providers – often in quick succession – in search of a quick solution, often resulting in contradictory diagnoses and incomplete treatment. Research conducted in the urban slum setting of Karachi, Pakistan, suggests that this pattern of ‘quick switches’ between providers results in poor health outcomes and even death [39].

In this context, the collective management of health information and health-related decisions is evident. People gain knowledge by word-of-mouth, and choices of where and at what moment to seek care are primarily informed by the advice of family and friends. There is little existing evidence about health information flow in urban slum communities, but the predominance of interpersonal information in health dissemination has been documented in rural areas of Bangladesh, where print and audio-visual media have shown little impact [38–42]. In our study population, this adds to the sense of uncertainty and confusion created by the fragmented provision of services, as contradictory recommendations are received. Decision making power is contingent with economic autonomy and decisions about care are taken considering their impact on competing household financial priorities. For the majority this means decisions are taken in collaboration with, or deferred to, other family members. This communal management of health can make accessing information and services for sensitive health issues difficult, particularly for girls and young women [43, 44].

Other notable barriers to the use of the formal healthcare system are perceptions of poor quality care [22, 25, 29], expressed as mistrust of health workers and experiences of poor treatment and corruption [45]. Conversely the main incentive to seek care from a particular provider aside from affordability was good quality care, defined as quick and respectful treatment and a fast recovery [46]. To better meet the needs of this population, access to comprehensive services including effective medication, diagnostic tests, and secondary level care, provided by respectful and trustworthy health workers are essential [8, 47, 48].

### Limitations

Whilst our sampling strategy included two groups (factory workers, and women and girls), in practice many of the women and girls interviewed were also factory workers. As a result, the distinction between the two groups was not as clearly delineated as we had originally hoped. It was explained to participants at the outset that their views would be drawn out with reference to the specific recruitment group, however we found that there was significant overlap in content between the two groups.

Additionally, as is the nature of qualitative research our results provide an in-depth contextualized understanding of health seeking behaviour in Kamrangirchar and Hazaribagh but are not necessarily generalisable; however we believe emergent concepts may be transferable to other similar settings.

## Conclusions

Residents of Kamrangirchar and Hazaribagh see their health as a necessary asset to maintaining an income, and the ability to work is a key driver for staying healthy. At the same time, staying healthy with very limited resources, demanding working hours, and in poor living and working conditions is difficult.

People expressed preference for a combination of rest and comprehensive allopathic care, yet resource barriers and the need to keep working often prevent this; a quick fix at the local pharmacy is deemed ‘good enough’ for the majority of ‘common’ health issues. When this fails, or a condition is deemed ‘serious’ the population is faced with an array of highly fragmented services. These are navigated in a process of ‘trial and error’ as opposed to effective early results, as people search for a quick solution, which will restore their ability to work.

Information about health and care is exchanged through social and family networks, decisions are shaped by collective understanding of illness and perceptions and experiences of appropriate care. For those with little economic autonomy or social capital, responsibility for decision-making rests with influential family members. This communal management of ill-health, combined with inconsistent service provision, means that experiences of care are often characterized by uncertainty, confusion, and ultimately lead to prolonged or unresolved health issues.

The findings of this study suggest that in Kamrangirchar and Hazaribagh, there is an acute need for access to effective and comprehensive healthcare services adapted to the needs of this vulnerable population. A new patient-centered model of care should include the provision of a complete package of affordable services, including accessible facilities providing both primary and secondary level care ‘under one roof’; the availability of diagnostic tests and medication; a strong referral and follow-up system including primary care and public, private and NGO-run specialised services; services adapted to the specific constraints faced by workers in accessing care; and respectful and attentive treatment by health workers. Health education should focus on working with communities to identify key areas of concern and utilising informal networks to support information sharing and decision making. Reaching particularly vulnerable groups such as girls and young women will require an approach targeting those who they turn to for health information and decision making.

Regarding unregulated pharmacy practices, which we saw described as playing a major role in the provision of care, further research and attention is urgently needed, particularly in the context of global concerns regarding antimicrobial resistance. It is essential that these issues are incorporated into strategies and interventions aiming to improve care for Dhaka’s slum residents, with rigorous documentation and evaluation to fill information gaps and inform policy and governance adaptation (Appendix). An approach that is specifically tailored to the needs of an urban slum context is necessary to improve healthcare for its population, which is particularly pressing as urban working populations continue to grow exponentially and slums become the dominant type of human settlement in many global cities.

### Additional file

Additional file 1: Topic guide for in-depth interviews. (PDF 24 kb)

## Appendix 1

### Implications for policy and practice: recommendations for further exploration

- Improve access to comprehensive primary and secondary level care for people living in Kamrangirchar and Hazaribagh, adapted to the specific needs and access constraints of workers.
- Map services and increase collaboration and partnership between healthcare providers, including establishing referral and patient follow-up systems.
- Increase regulation and improve knowledge of pharmacists to ensure accurate dispensing and referral.
- Work with communities to identify information gaps and tailor information and messaging to the questions people have.(.including self-treatment; appropriate therapeutic regimens; which provider to go to for what; how to identify quality medication etc).
- Increase health-related knowledge and skills to facilitate decision-making, creating more informed patients in the longer term and increasing the demand for higher quality of care (for inappropriate antibiotics and injections, for example).
- Utilise preferences for word-of-mouth communication and informal networks to support information sharing and decision making; develop creative mechanisms to reach harder to access groups such as young women and girls (e.g. using community ‘ambassadors’ and community-based health education programmes).

## Abbreviations

CIPRB: Centre for Injury Prevention and Research Bangladesh
FFW: Female factory worker
HW: Housewife
icddr,b: International Centre for Diarrhoeal Disease Research, Bangladesh
KI: Key informant
MBBS: Bachelor of Medicine, Bachelor of Surgery
MFW: Male factory worker
MSF: Médecins Sans Frontières
NGO: Non-governmental organisation

## Footnotes

1 Please note: since the time of writing the Hazaribagh tanneries have been relocated to a new tannery industrial complex in Savar, in the northwest of Dhaka City.

2 100 BDT is 1.20 USD. The minimum wage in Bangladesh is 1500 taka a month, or approximately 18 USD (although some economic sectors have an industry-specific minimum wage such as the garment sector, which is officially 5300 taka or 68 USD).
