# Supplementary material for: ‘Working to stay healthy’, health-seeking behaviour in Bangladesh’s urban slums: a qualitative study
Source: BMC Public Health. 2019 May 17;19:600. doi: 10.1186/s12889-019-6750-0 (PMC6525448; doi:10.1186/s12889-019-6750-0)
Supplement: Supplementary file 1 — Topic guide for in-depth interviews. (PDF 24 kb) [file 12889_2019_6750_MOESM1_ESM.pdf]

SAMPLE TOPIC GUIDE: HEALTH SEEKING BEHAVIOUR

Introductions

- Introductions
- Explain purpose, length, what we ask of participants
- Any questions before we begin?

Topic 1: Life history

- Grand opening question: To begin with, tell me about yourselves and your life here?
    - Tell us about your family / living situation / work / social life?
    - How do you feel about life here?

Topic 2: Perceptions of health

- Tell us about how you see the health situation here in your community…
    - What are the issues? Why?
    - Has this changed over time? How? Why?
- How do you know if you are healthy or sick?

Topic 3: Experiences of sickness and care seeking

- Tell us about the last time you were sick; what did you do?
    - Why did you go there/do that?
    - Who was involved in the decision to go there/do that?
    - Tell us about your experience there?
    - If it was good/bad care, why?
- What options do people have here when they're unwell?
    - Why do people choose these options?
    - What influences their decisions?
- What barriers do people face getting care?
- What facilitates people getting care?

Topic 4: Health information

- Where do people get information about health and health services?
    - Why?
- What messages to people get about health and healthcare?
    - How?
    - What impact does this have?
- What information do people need / what questions do people have about health and care?

Topic 5: Looking to the future…

- Tell us how you think healthcare can be improved here?
- Tell us what you think are the best ways to provide people with information about their health/health services?

Topic 6: Concluding the interview

- Of everything we discussed today, what has been most important for you?
- Is there anything you would like to add to this discussion, or anything that we missed?
- Do you have any questions you would like to ask me?
- Thanks and close
